# Antibiotic exposure in the first two years of life and development of asthma and other allergic diseases by 7.5 yr: A dose-dependent relationship

**DOI:** 10.1111/pai.12153

**Published:** 2013-12-02

**Authors:** Lauren Hoskin-Parr, Alison Teyhan, Ariel Blocker, A J W Henderson

**Affiliations:** 1 School of Cellular and Molecular Medicine, University of BristolBristol, UK; 2 School of Social and Community Medicine, University of BristolBristol, UK

**Keywords:** Avon Longitudinal Study of Parents and Children, antibiotics, atopy, childhood asthma, eczema, hay fever, wheeze

## Abstract

**To cite this article:** Hoskin-Parr L, Teyhan A, Blocker A, Henderson AJW. Antibiotic exposure in the first two years of life and development of asthma and other allergic diseases by 7.5 yr: a dose-dependent relationship. *Pediatr Allergy Immunol* 2013: **24:** 762–771.

**Background:**

Antibiotic use in infancy disrupts gut microflora during a critical period for immune system development. It is hypothesized that this could predispose to the development of allergic diseases. We investigated the associations of antibiotic use in the first 2 yr of life with the development of asthma, eczema or hay fever by age 7.5 yr in a longitudinal birth cohort.

**Methods:**

Subjects were 4952 children from the Avon Longitudinal Study of Parents and Children (ALSPAC). Child antibiotic use and asthma, eczema and hay fever symptoms were maternally reported. Atopy was assessed by skin prick tests at age 7.5 yr. The total number of antibiotic courses was considered as the main exposure. Data were analysed using multivariate logistic regression.

**Results:**

Children reported to have taken antibiotics during infancy (0–2 yr) were more likely to have asthma at 7.5 yr (OR 1.75, 95% CI 1.40–2.17), and the odds (OR, [95% CI]) increased with greater numbers of courses: once 1.11 [0.84–1.48]; twice 1.50 [1.14–1.98]; three times 1.79 [1.34–2.40]; four times or more 2.82 [2.19–3.63]. Increased antibiotic use was also associated with higher odds of eczema and hay fever but not atopy. The effect appeared to be associated with cumulative rather than a critical period of exposure during the first 2 yr.

**Conclusions:**

A robust and dose-dependent association was found between antibiotic use in the first 2 yr of life and asthma at age 7.5 yr but did not appear to be mediated through an association with atopy.

Worldwide asthma prevalence has risen considerably over the last 30 yr (1), particularly in developed, Western countries (2). With increasing global urbanization, asthma prevalence is predicted to increase further (3). Although asthma has a large heritable component, the timescale of the increase in asthma prevalence suggests environmental influences. One factor that has received considerable attention in this regard is exposure to and variations in host responses to the microbial environment (4).

Increased prevalence of asthma and allergic diseases has coincided with an increase in antibiotic prescriptions in developed countries. Antibiotics in infancy cause disruption to the gut microflora at a time of critical immune development, which could predispose to the development of an allergic phenotype, manifesting as asthma and other common childhood allergic diseases. However, although many studies have investigated the association between antibiotics and asthma in early childhood, the question of causality remains unresolved. Positive associations between reported antibiotic use in infancy and later asthma have been reported (5–10) but not consistently replicated (11–19). Inconsistencies could be explained in part by methodological limitations, such as cross-sectional studies with a high risk of recall bias in reported antibiotic use (20) or studies that could not take account of potential reverse causation, where antibiotics have been prescribed for early symptoms of allergic diseases (14).

In this study, we evaluated the association between timing and number of courses of infant antibiotic exposure and the development of asthma and allergic diseases in a birth cohort study that collected information on antibiotic use in infancy on relevant outcomes, including objective tests of allergic sensitization, in later childhood. Additionally, the availability of symptom reports in infancy allowed us to test for reverse causation of antibiotic prescription for allergic symptoms.

## Materials and methods

### Study population

Subjects were participants in the Avon Longitudinal Study of Parents and Children (ALSPAC), a population-based cohort that recruited 14,541 expectant mothers with due dates between 1 April 1991, and 31 December 1992, in Avon, United Kingdom. Data about the children were collected approximately annually using questionnaires sent to their mothers from age 6 months onwards and, from age 7 yr, in research clinics. Details of the study protocol have been published (21), and further information can be found at http://www.bris.ac.uk/alspac. Parents gave written consent, and the study was approved by the ALSPAC Ethics and Law Committee and the Local Research Ethics Committees. Please note that the study website contains details of all the data that is available through a fully searchable data dictionary; <http://www.bris.ac.uk/alspac/researchers/data-access/data-dictionary/>.

### Exposure

Mothers reported whether their child had taken antibiotics (‘No’, ‘Yes, once’ or ‘Yes, twice or more’) in three questionnaires covering the periods 0–6, 6–15 and 15–24 months. Three exposure variables were derived as follows: any antibiotic use during the period 0–24 months; the total number of times antibiotics had been taken between 0 and 24 months (‘None’, ‘Once’, ‘Twice’, ‘Three times’ or ‘Four+ times’); and a variable indicating when antibiotics had been taken (‘None’, ‘0–6 months only’, ‘6–15 months only’, ‘15–24 months only’, ‘0–6 and 6–15 months’, ‘0–6 and 15–24 months’, ‘6–15 and 15–24 months’, and ‘0–6, 6–15 and 15–24 months’). As questionnaire responses included 2 or more antibiotic courses as the highest possible category, there was uncertainty about the precise number of courses taken over the three surveys. We addressed this by performing a sensitivity analysis, allocating all ‘2 or more’ responses to the highest category (four+ times). Although this would lead to misclassification of some subjects from lower to higher frequency, this would have the effect of attenuating differences between the lowest and highest category, resulting in a conservative estimate of the effect size.

### Outcomes

The primary outcome was asthma at age 91 months (approximately 7.5 yr) defined as maternal report of a physician's diagnosis of asthma at any time and symptoms of wheezing during the previous 12 months. Two secondary outcomes, eczema and hay fever, were maternally reported at the same time. Children were also invited to attend a research clinic at 7.5 yr, at which atopy was determined by skin prick testing and defined as a positive response (≥2 mm weal) to any one of *Dermatophagoides pteronyssinus*, grass or cat allergen with a negative response to diluent solution. As previously reported, this definition identified >95% of subjects with any positive response to a wider panel of allergens (22). To test for reporting bias, we considered reported headache at 91 months, for which there is no plausible link with antibiotic use in infancy, as a positive control. As we did not have direct reports of indication for antibiotic prescriptions, we tested for reverse causation by excluding from analysis infants with any reported wheezing from birth to 18 months, and from birth to 30 months, for which they may have received antibiotics. These two time periods were chosen due to lacking the necessary data to exclude children exclusively up to 24 months. Hence, we advise that these values are viewed as conservative.

### Confounders

Potential confounders of the association between antibiotic use in infancy and allergy at age 7.5 yr were selected on the basis of prior reports or theoretical grounds and were grouped in four categories: child-based variables included sex, ethnicity and age of child at time of outcome; birth-related variables included mother's age at time of delivery, birth mode, birthweight and gestation; socioeconomic status was assessed by marital status, home ownership status, mother's highest educational qualification and degree of difficulty in paying for food; lifestyle variables included breastfeeding, time spent outdoors by the child, disinfectant use by mother during pregnancy as a measure of hygiene, mother's smoking during pregnancy and child's contact with cats.

### Statistical analysis

Associations between each antibiotic use exposure variable and each outcome were tested using logistic regression in unadjusted analyses followed by adjusted models taking account of possible confounding variables. These were added to the model in a hierarchical order based on the groups described above; child-based variables followed by birth variables, socioeconomic variables and lifestyle variables. The analysis for asthma was repeated after exclusion of children with a reported history of early wheezing. To test whether the association between antibiotic use and any of the outcomes differed for boys and girls, interaction terms between the exposure variables and child sex were fitted. No strong evidence for interaction was found so results unstratified by sex are presented. Statistical analysis was conducted using STATA v12 (StataCorp, College Station, TX, USA).

## Results

**Figure 1:**
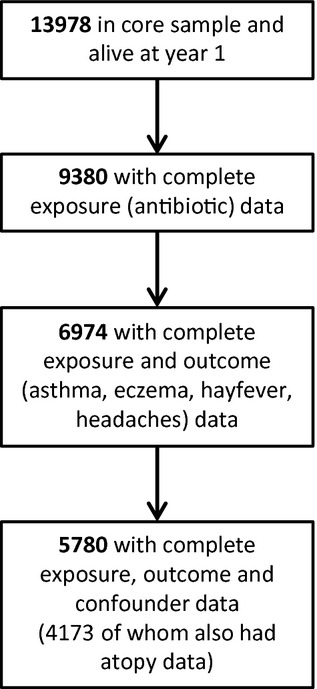
Flow diagram showing derivation of study population who had complete data on exposures, outcomes and confounders.

Of 13,978 live born children surviving to at least 1 yr, complete data on antibiotic use in infancy, outcomes and confounders were available for 5780 children at age 7.5 yr, and of those, 4173 also had skin prick sensitivity data (Fig. 1). Children with missing data were more likely than those with complete data to be of non-white ethnicity, born preterm and with low birthweight and to have mothers who were younger, living in rented accommodation and had lower educational qualifications. Children with missing data were also more likely to have received two or more courses of antibiotics during the first 24 months (Table 1).

**Table 1:** Comparison between children in the study population (who had complete data on all exposures, outcomes and confounders) and those with incomplete data

Variable	Study population N = 5780 (%)	Incomplete N differs by variable (%)
Sex		N = 8198
Girl	48.8	48.1
Child's ethnic group*		N = 6303
White	96.8	93.3
Child's age-outcome questionnaire*		N = 2382
≤91 months	67.3	54.7
Mother's age at delivery (%)*		N = 5968
≤20	1.9	10.9
21–25	15.9	28.9
26–30	43.8	36.1
31–35	29.8	18.4
≥36	8.6	5.8
Mode of birth		N = 5968
Caesarean	10.1	11.0
Birthweight*		N = 8018
Normal (≥2500 g)	96.3	93.4
Gestation time*		N = 8198
Normal (≥37 wk)	95.5	92.9
Child breastfed*		N = 5547
≥6 months	34.7	22.5
<6 months	46.0	48.0
Never	19.3	29.6
Marital status of mother*		N = 7307
Married	84.4	67.4
Home ownership status*		N = 7247
Mortgaged/Owned	85.5	63.7
Privately rented	5.1	8.9
Council rented	7.3	22.9
Other	2.2	4.5
Mother's highest qualification*		N = 6638
Degree	17.7	8.7
A-level	27.2	18.5
Below A-level	55.1	72.9
Difficulty in paying for food*		N = 2523
Not difficult	97.1	95.1
Difficult	2.9	4.9
Antibiotics 0–6 months*		N = 5653
No	69.1	66.5
Yes, only once	22.2	21.7
Yes, twice or more	8.7	11.9
Antibiotics 6–15 months		N = 5240
No	46.8	45.7
Yes, only once	26.8	26.0
Yes, twice or more	26.5	28.3
Antibiotics 15–24 months*		N = 4605
No	54.0	50.6
Yes, only once	25.9	24.3
Yes, twice or more	20.2	25.2
Asthma in past 12 months*		N = 2410
Yes	10.9	13.4
Doctor has ever said child has asthma		N = 2351
Yes	19.3	23.2
Eczema in past 12 months*		N = 2406
Yes	17.2	14.3
Hay fever in past 12 months		N = 2386
Yes	8.7	8.9
Sensitization to allergens†	N = 4173	N = 2453
Yes	19.9	21.2
Early wheeze 0–6 months*		N = 5677
Yes	19.0	24.2
Early wheeze 6–18 months*		N = 5276
Yes	20.1	23.5
Time child spends outdoors per week*		N = 4541
≥7 h	78.3	74.5
Maternal disinfectant use during pregnancy*		N = 7347
<Once a week	37.3	31.1
Once a week	37.4	33.9
>Once a week	25.3	35.0
Maternal smoking in first 3 months of pregnancy*		N = 7378
No	16.4	31.8
Child had weekly contact with cats at 24 months		N = 4642
No contact	45.3	41.9

* p < 0.05 in Chi-squared test.

† Skin sensitisation (atopy) data only available for 4173 children in the study population.

Of the 5780 children with complete data availability, 3992 (69.1%) were not given antibiotics between the age of 0 and 6 months, 2702 (46.8%) were not given antibiotics between 6 and 15 months and 3119 (54.0%) were not given antibiotics between 15 and 24 months. At 7.5 yr, 617 (10.7%) had asthma, 992 (17.2%) had eczema, 504 (8.7%) had hay fever and 829 (19.9%) of those with skin prick test data were atopic. During the periods 0–6 and 6–18 months, respectively, 1100 (19.0%) and 1159 (20.1%) subjects reported wheeze. Compared with boys, girls had lower odds (OR, [95% CI]) of asthma (0.70, [0.59–0.83]) and hay fever (0.63, [0.53–0.77]), but greater odds of eczema (1.21, [1.05–1.39]). Children of non-white ethnic origin were more likely than white children to have an allergic disease (OR, [95% CI] 1.56, [1.03–2.35] for asthma; 1.43, [1.01–2.04] for eczema; 1.64, [1.05–2.55] for hay fever).

Children who had taken antibiotics by 24 months were more likely to have asthma by age 91 months compared with those that had not (OR, [95% CI] 1.75, [1.40–2.17]), with a greater effect for increasing number of courses: (OR [95% CI] 1.11, [0.84–1.48]) for antibiotics once, 1.50, [1.14–1.98] for antibiotics twice, 1.79, [1.34–2.40] for antibiotics three times and 2.82, [2.19–3.63] for antibiotics four times or more (Table 2). There was little evidence of attenuation of the effect size by any of the confounding variables considered in the fully adjusted model (Table 3). When the analysis was restricted to subjects without symptoms of early wheeze before the age of 18 months (n = 4042, 312 [7.7%] of whom had asthma at 7.5 yr), the associations were substantially attenuated (OR (95% CI) 0.90, [0.65–1.26], 0.89, [0.62–1.27], 1.25, [0.86–1.83], and 1.65, [1.16–2.35], respectively, for antibiotics taken once, twice, three times, and four times or more) (Table 2). Restriction to those without wheeze before 30 months (n = 3602, 6.1% of whom had asthma at 7.5 yr) attenuated associations further (Table 2).

**Table 2:** Fully adjusted analysis of all outcomes (asthma, asthma with no early wheeze, eczema, hay fever, headaches and sensitization) against each exposure variable (any vs. none, 5 level and 8 level)

Outcome	Antibiotics taken					
	Any vs. none		Testing dose dependency (5 level variable)		Testing time dependency (8 level variable)	
Asthma	No	Ref	None	Ref	None	Ref
	Yes	1.75 (1.40–2.17)	Once	1.11 (0.84–1.48)	0–6 months only	1.12 (0.72–1.74)
			Twice	1.50 (1.14–1.98)	6–15 months only	1.13 (0.81–1.56)
			Three times	1.79 (1.34–2.40)	15–24 months only	1.50 (1.08–2.07)
			Four times or more	2.82 (2.19–3.63)	0–6 and 6–15 months	1.52 (1.07–2.16)
					0–6 and 15–24 months	1.59 (0.97–2.60)
					6–15 and 15–24 months	2.13 (1.63–2.78)
					0–6, 6–15 and 15–24 months	2.60 (1.98–3.42)
Asthma no early wheeze (before 18 months) (n = 4042)	No	Ref	None	Ref	None	Ref
	Yes	1.09 (0.84–1.41)	Once	0.90 (0.65–1.26)	0–6 months only	0.90 (0.50–1.61)
			Twice	0.89 (0.62–1.27)	6–15 months only	0.82 (0.55–1.22)
			Three times	1.25 (0.86–1.83)	15–24 months only	1.07 (0.72–1.59)
			Four times or more	1.65 (1.16–2.35)	0–6 and 6–15 months	0.89 (0.53–1.51)
					0–6 and 15–24 months	0.96 (0.47–1.98)
					6–15 and 15–24 months	1.33 (0.95–1.86)
					0–6, 6–15 and 15–24 months	1.46 (0.97–2.19)
Asthma no early wheeze (before 30 months) (n = 3602)	No	Ref	None	Ref	None	Ref
	Yes	0.90 (0.67–1.20)	Once	0.85 (0.59–1.24)	0–6 months only	0.91 (0.47–1.75)
			Twice	0.71 (0.46–1.09)	6–15 months only	0.79 (0.51–1.23)
			Three times	0.97 (0.61–1.54)	15–24 months only	0.94 (0.59–1.49)
			Four times or more	1.24 (0.80–1.91)	0–6 and 6–15 months	0.67 (0.35–1.28)
					0–6 and 15–24 months	0.96 (0.40–2.30)
					6–15 and 15–24 months	1.03 (0.69–1.55)
					0–6, 6–15 and 15–24 months	0.94 (0.56–1.60)
Eczema	No	Ref	None	Ref		
	Yes	1.20 (1.02–1.41)	Once	1.05 (0.85–1.29)		
			Twice	1.23 (1.00–1.51)		
			Three times	1.17 (0.93–1.47)		
			Four times or more	1.41 (1.14–1.74)		
Hay fever	No	Ref	None	Ref		
	Yes	1.28 (1.03–1.60)	Once	1.17 (0.88–1.54)		
			Twice	1.21 (0.90–1.61)		
			Three times	1.18 (0.86–1.61)		
			Four times or more	1.60 (1.21–2.10)		
Headache	No	Ref	None	Ref		
	Yes	1.18 (1.05–1.33)	Once	1.12 (0.96–1.30)		
			Twice	1.21 (1.04–1.42)		
			Three times	1.17 (0.99–1.39)		
			Four times or more	1.23 (1.05–1.45)		
Sensitization	No	Ref	None	Ref		
	Yes	1.02 (0.85–1.22)	Once	1.00 (0.80–1.25)		
			Twice	1.10 (0.88–1.39)		
			Three times	0.96 (0.74–1.24)		
			Four times or more	1.00 (0.79–1.27)		

Models were fully adjusted for child-based variables (sex, ethnicity and age of child at time of outcome), birth-related variables (mother's age at time of delivery, birth mode, birthweight and gestation), socioeconomic status (marital status, home ownership status, mother's highest educational qualification and degree of difficulty in paying for food) and lifestyle variables (child breastfed, time spent outdoors by the child, disinfectant use by mother during pregnancy, mother's smoking during pregnancy and child's contact with cats).

**Table 3:** Five model multivariate analysis of ‘Asthma no early wheeze up to 18 months’ and ‘5 level antibiotic variable’, showing the addition of covariates to build on each model

Outcome: asthma, no early wheeze		Odds ratio (95% confidence intervals)				
Variable	Categories	Model 1	Model 2	Model 3	Model 4	Model 5
Number of times antibiotics taken	None	Ref	Ref	Ref	Ref	Ref
	Once	0.91 (0.66–1.27)	0.91 (0.65–1.26)	0.90 (0.64–1.25)	0.90 (0.65–1.26)	0.90 (0.65–1.26)
	Twice	0.88 (0.62–1.26)	0.87 (0.61–1.24)	0.86 (0.61–1.23)	0.88 (0.62–1.26)	0.89 (0.62–1.27)
	Three times	1.27 (0.88–1.85)	1.27 (0.88–1.85)	1.24 (0.85–1.81)	1.25 (0.86–1.82)	1.25 (0.86–1.83)
	Four times or more	1.73 (1.22–2.45)	1.67 (1.18–2.36)	1.66 (1.17–2.36)	1.66 (1.17–2.37)	1.65 (1.16–2.35)
Sex	Male		Ref	Ref	Ref	Ref
	Female		0.77 (0.61–0.97)	0.76 (0.60–0.96)	0.75 (0.59–0.95)	0.75 (0.59–0.95)
Ethnicity	White		Ref	Ref	Ref	Ref
	Non-White		1.78 (1.07–2.98)	1.68 (1.00–2.83)	1.61 (0.95–2.74)	1.61 (0.95–2.75)
Age of child at time of outcome questionnaires	≤91 months		Ref	Ref	Ref	Ref
	≥92 months		0.98 (0.76–1.26)	0.99 (0.77–1.27)	0.99 (0.77–1.27)	0.99 (0.77–1.27)
Mothers age at time of delivery	≤20 yr			0.78 (0.30–1.98)	0.77 (0.29–2.01)	0.76 (0.29–1.99)
	21–25 yr			1.08 (0.77–1.50)	1.04 (0.74–1.47)	1.03 (0.73–1.45)
	26–30 yr			Ref	Ref	Ref
	31–35 yr			0.90 (0.68–1.19)	0.90 (0.68–1.20)	0.92 (0.69–1.22)
	≥36 yr			0.88 (0.57–1.37)	0.90 (0.58–1.39)	0.93 (0.60–1.45)
Birthweight	Normal birthweight			Ref	Ref	Ref
	Low birthweight			3.38 (2.04–5.60)	3.33 (2.00–5.56)	3.31 (1.98–5.53)
Gestation	37 wk or longer			Ref	Ref	Ref
	Preterm			0.47 (0.23–0.93)	0.47 (0.24–0.94)	0.46 (0.23–0.92)
Birth mode	Natural			Ref	Ref	Ref
	Caesarean			1.30 (0.91–1.86)	1.31 (0.91–1.87)	1.27 (0.89–1.83)
Marital status	Married				Ref	Ref
	Not married				0.84 (0.59–1.20)	0.88 (0.61–1.26)
Home ownership status	Owned/Mortgaged				Ref	Ref
	Privately Rented				1.56 (0.97–2.51)	1.56 (0.97–2.52)
	Council rented				1.61 (1.03–2.51)	1.60 (1.02–2.51)
	Other				0.92 (0.36–2.32)	0.93 (0.37–2.36)
Mother's highest qualification	Degree				Ref	Ref
	A-level				0.93 (0.65–1.33)	0.88 (0.61–1.26)
	CSE/vocational/O level				0.89 (0.64–1.24)	0.80 (0.57–1.14)
Degree of difficulty in paying for food	No				Ref	Ref
	Yes				1.70 (0.94–3.06)	1.71 (0.95–3.09)
Child breastfed	≥6 months					Ref
	<6 months					1.25 (0.95–1.66)
	Never					1.29 (0.90–1.86)
Time spent outdoors by child	≥7 h					Ref
	≤6 h					1.01 (0.76–1.34)
Disinfectant use during pregnancy	<Once per week					Ref
	Once per week					1.20 (0.92–1.58)
	>Once per week					1.01 (0.73–1.38)
Mother's smoking during pregnancy	No					Ref
	Yes					0.98 (0.70–1.37)
Child's contact with cats	No					Ref
	Yes					0.91 (0.71–1.15)

Model 1: No co-variables.

Model 2: Child-based variables (sex, ethnicity and age of child at time of outcome).

Model 3: Child-based variables and birth-related variables (mother's age at time of delivery, birth mode, birthweight and gestation).

Model 4: Child-based variables, birth-related variables and socioeconomic status (marital status, home ownership status, mother's highest educational qualification and degree of difficulty in paying for food).

Model 5: Child-based variables, birth-related variables, socioeconomic status and lifestyle variables (child breastfed, time spent outdoors by the child, disinfectant use by mother during pregnancy, mother's smoking during pregnancy and child's contact with cats).

In a sensitivity analysis, 1168 children were reclassified from the ‘twice’ and ‘three times’ categories to the highest (four+ times) category. This made little difference to the effect estimates (OR (95% CI) 1.11, [0.84–1.48], 1.39, [1.00–1.94], 1.82, [1.02–3.25] and 2.23, [1.77–2.81], respectively, for antibiotics taken once, twice, three times, and four times or more).

Children who had taken antibiotics during any one time period were more likely to have asthma by age 91 months than those who had not; 0–6 months 1.12, [0.72–1.74]; 6–15 months 1.13, [0.81–1.56]; 15–24 months 1.50, [1.08–2.07]. The effect sizes for those who had taken antibiotics in two time periods were greater than for those who had only taken them in one time period. The strongest association was seen for children who had taken antibiotics in all three time periods 2.60, [1.98–3.42]. Associations were attenuated when the analysis was restricted to subjects without early wheeze (Table 2).

Compared with asthma, weaker effect sizes were found for hay fever and eczema although with the same direction of association (Table 2). Compared with those who had never taken antibiotics before the age of 24 months, those who had taken them had an OR (95% CI) of 1.28, [1.03–1.60] for hay fever and 1.20, [1.02–1.41] for eczema. There was no association between infant antibiotic use and atopy on skin prick testing at 7.5 yr or reported headaches at the same age (Table 2).

## Discussion

Our results show strong, dose-dependent associations between antibiotics in infancy and asthma in later childhood. An association was evident but weaker with eczema and hay fever. These associations were not attenuated by adjustment for confounders. However, there was evidence that reverse causation explained part of the association with asthma as exclusion of children with early wheeze up to 30 months, for which they may have received antibiotics, weakened the association substantially. Therefore, inappropriate treatment for young children for symptoms of wheezing with antibiotics could result in a spurious association between antibiotics and later asthma. There was no strong evidence of association with headaches indicating that health reporting was not likely to explain the results. In contrast to reported symptoms, infant antibiotic use was not positively associated with skin test sensitization, indicating no objective evidence of association with atopy. Our analysis of timing and number of courses of antibiotics reported suggested that cumulative exposure may have been more important than a single critical period effect although our ability to resolve the latter was limited by the fixed periods covered by successive questionnaires.

The literature to date has been conflicting, making it hard to draw any firm conclusions about the likely causal role of early antibiotic use on the risk of asthma and allergy in childhood. Several studies have reported a strong association between antibiotic use in infancy and later development of asthma (5–9). Some of these were retrospective studies, with data on antibiotic use dependent on maternal recall up to 6 yr later, which is likely to introduce recall bias (5, 6, 8). However, longitudinal cohort studies have also described positive associations of asthma with antibiotic use in infancy ascertained prospectively (7, 8), suggesting that recall bias alone is insufficient to explain this relationship.

Due to the nature of data collection, many studies have been unable to document indications for antibiotic prescription in infancy. Where this has been possible, results suggest that reverse causation or confounding by indication could explain a large part of the reported association (15–19). Rusconi et al. (19) in 2011 reported an association between antibiotics and early asthma but not with late onset asthma, implying reverse causation. In a longitudinal study of over 4000 subjects, Celedon et al. (14) in 2004 reported no association between antibiotic use in the first year of life and asthma development between the ages of 2 and 5 yr when adjusted for lower respiratory illnesses in the first year, indicating confounding by indication. Their explanation that antibiotics were more frequently used in children with asthma symptoms in the first year is consistent with the attenuation of effects in the present study when children with early wheeze were excluded. Therefore, although we found an association between reported asthma and antibiotic use in infancy that appeared to be robust to confounding, we cannot completely discount the possibility suggested by Penders et al. (23) that this was likely to be explained by confounding by indication and reverse causation.

The putative explanation for an association between antibiotic use in infancy and later asthma is based on the hygiene hypothesis, whereby exposure to microbial products in early childhood, possibly through induction of type 1 T-helper (Th1) lymphocytes, diminishes Th2-biased responses characteristic of allergy. Studies of children growing up on farms [reviewed by Genuneit (24)] show reduced risks of asthma and allergy in later childhood compared with rural children not exposed to a farming environment. This may be explained by exposure to endotoxins interacting with genetic variants in genes for components of the innate immune system, such as toll-like receptors (25). The human gut microbiota may also have a role in altering risk of childhood asthma and allergy. Reduced microbial diversity in infant faecal samples has been associated with increased risk of allergic diseases in later childhood (26). Although it is suggested that these mechanisms are mediated by atopy, we did not find strong evidence in this study of a specific association of antibiotic use with atopy. This is consistent with reported associations of reduced gut microbial diversity in early infancy and the development of eczema but not atopy in a population of children at high risk of allergy (27). Therefore, in contrast to farming exposures where there is strong evidence of effects mediated through allergy, there may be other mechanisms that are important in mediating effects of alterations in gut microbiota, such as non-atopic inflammation. It is important to fully understand these in order to plan for effective interventions for primary prevention of this group of diseases. Current evidence suggests equivocal effects on clinical outcomes of attempts to promote protective gut microbiota with the use of probiotics. Therefore, the use of a single intervention strategy in the face of immunological complexity in the origins of allergic diseases has recently been called into question (28).

The main strength of our study was the use of a large longitudinal cohort, with prospective collection of exposure data and the ability to account for a wide range of confounding influences. We were also able to test for dose dependency and to look at whether the timing of antibiotics was linked to outcome. Additionally, because of the breadth of ALSPAC data, we were able to consider other symptoms (headaches) as a check for reporting bias. As all variables were collected within 12 months of the event, there was less likelihood of recall bias compared with other studies (5, 6, 20).

Our main limitation was reliance on maternal report of antibiotic use, which was not verified by medical records and which did not specify which antibiotics were used or their indication. Therefore, we were unable to consider confounding by indication. We considered reverse causation by excluding from analysis infants with wheezing in infancy for which antibiotics may have been prescribed. By relying on maternal report for both exposure and outcome, our results could be biased by general increased reporting of all health outcomes, including antibiotic use, in questionnaire responses. There is also evidence that a variety of drugs reported by mothers to be used during pregnancy, including antibiotics are associated with asthma risk in their children but the association is largely confounded by concomitant use of anti-asthma drugs by the mother (29). We attempted to account for reporting bias by mothers with asthma, who may be more likely to seek treatment for their children, by considering both reverse causation and by using an unrelated health outcome (headache) as a marker of general health reporting.

Asthma in this study was not confirmed by objective measurements, such as bronchial hyper-responsiveness, which has low prevalence in community-based samples. However, misclassification of the outcome is unlikely to be related to exposure reports, which were collected prospectively in infancy. Therefore, we believe such misclassification is likely to be random and would have the effect of attenuating our effect estimates rendering them conservative. A further limitation was that we were unable to categorize antibiotics by class. Jedrychowski et al. (30) reported that early use of macrolides and cephalosporins, two broad spectrum antibiotics, was strongly associated with increased risk of developing asthma at 5 yr of age. In contrast, there is evidence that macrolide antibiotics could increase symptom-free days in children following exacerbations of asthma, possibly through anti-inflammatory effects rather than perturbation of the human microbiome (31). It is likely in UK practice that broad spectrum antibiotics would have been utilized in this population at the time (early 1990s). Although we were able to account for a large number of possible confounders, we cannot discount the possibility of residual confounding although there was strikingly little attenuation with adjustment for confounders that we would have predicted to be associated with health seeking behaviours and reporting.

In common with many longitudinal, population-based studies, there was substantial loss to follow-up resulting in missing outcome data in a proportion of the sample. There was a suggestion that subjects with missing outcome data had more courses of antibiotics in infancy. If there also was differential loss to follow-up of children without asthma, there may have been inflation of effect estimates in the complete case analysis.

## Conclusion

We have demonstrated a strong, dose-dependent association between antibiotic use in infancy and later asthma. However, the lack of association with objectively measured atopy is contradictory to the putative mechanism of association through pressure on the developing immune system towards an atopic phenotype. We did find attenuation of associations when we accounted for reverse causation but evidence of a positive and dose-dependent association remained. Confirmation of these findings requires a focus on non-atopic asthma in childhood in a longitudinal study with validated antibiotic prescription data and documentation of indications for antibiotic use in infants.
